# Feasibility of shortened scan acquisition time with IQ-SPECT technology using SMARTZOOM(TM) collimator in myocardial perfusion imaging

**DOI:** 10.22038/aojnmb.2025.78149.1551

**Published:** 2025

**Authors:** Saranya Thiruvengadam, Dinesh Kumar Gauthaman, Nikita Sampathirao, Indirani Elangovan, Shelley Simon

**Affiliations:** Department of Nuclear Medicine, Apollo Hospitals, Chennai, India

**Keywords:** IQ-SPECT, Cardiac-specific SMARTZOOM (TM) collimator, Myocardial perfusion imaging Shorter acquisition time, Image quality

## Abstract

**Objective(s):**

IQ-SPECT technology uses cardiac-specific SMARTZOOM(TM) collimator that can reduce the time required to acquire myocardial perfusion images to half (14 seconds/view) of a standard SPECT procedure. This study aimed to further shorten the scan acquisition time (7 seconds/view) using the SMARTZOOM(TM) collimator without compromising the image quality.

**Methods:**

This prospective observational study involved 50 patients (39 men and 11 women) who underwent myocardial perfusion studies using the SMARTZOOM(TM) collimator. The scans were acquired in 7 seconds/view (Group A) and 14 seconds/view (Group B). Attenuation-corrected (AC), non-attenuation-corrected (NAC), and polar maps were generated for both groups using the QGS/QPS software. The groups were qualitatively and quantitatively compared in terms of image quality, relative uptake score, summed scores, total perfusion deficit, and left ventricular ejection fraction (LVEF).

**Results:**

The image quality of both groups was comparable in the AC studies, whereas Group B was superior to Group A in the NAC studies. All images exhibited an image quality score of ≥3, which suggested adequate image quality. The mean LVEF values were 62.16 and 64.34 in Groups A and B, respectively (p-value 0.326). A strong positive correlation was observed between the two datasets (Pearson’s r=0.59). The mean summed score in the AC images was 7.5 in both groups (p-value 0.49), and in the NAC images, the scores were 7.68 in Group A and 7.46 in Group B (p-value 0.46). The mean total perfusion deficits calculated using the 17-segment models were 11% and 16% in Group A and Group B, respectively, for the AC images (p-value 0.143) and 11.2% and 16.5% in Group A and Group B, respectively, for the NAC images (p-value 0.135). Significant differences were not noted in the calculation of the relative uptake score in segments 1– 17 for the AC and NAC images in Groups A and B.

**Conclusion:**

The findings from this study indicate that further shortening of the scan acquisition time to 7 seconds/view is possible in myocardial perfusion imaging using the SMARTZOOM(TM) collimator without compromising the scan quality and the results, thus improving patient comfort.

## Introduction

 Myocardial perfusion SPECT imaging is routinely performed in most nuclear medicine departments globally. Cardiac nuclear imaging has evolved over the years, with modern advances in imaging equipment, techniques, and protocols to produce excellent-quality images. Qualitative and quantitative analysis of the information thus obtained can be used to diagnose a broad spectrum of cardiac diseases (1). A major utility of myocardial perfusion imaging (MPI) is the evaluation of widely prevalent

coronary artery disease (CAD). The ability of this technique to reflect myocardial perfusion during rest and stress and the wide range of quantitative data acquired from the gating of the study have made it a convenient diagnostic modality that is readily available and helps clinicians in making decisions in the management of CAD.

 A significant development in MPI is the advancement in hardware technology. Highly sensitive detectors and cardiac-specific dedicated collimators with a geometry focused on the heart and capturing four times more counts than the conventional low-energy high-resolution (LEHR) parallel hole collimators are now available (2). Conventional parallel whole collimators are not ideal for small organs as they require a prolonged scan time and also high injected doses to acquire an optimum number of counts for good image quality. Furthermore, multiple factors such as detector motion, size and shape of the collimator holes, deflections of the gantry, and distance between the patient and the detector affect the overall image quality. In addition, the rotational movement of the gantry with the detectors in close proximity to the patient for a long duration might be an additional factor of concern in those with claustrophobia.

**Figure 1:**
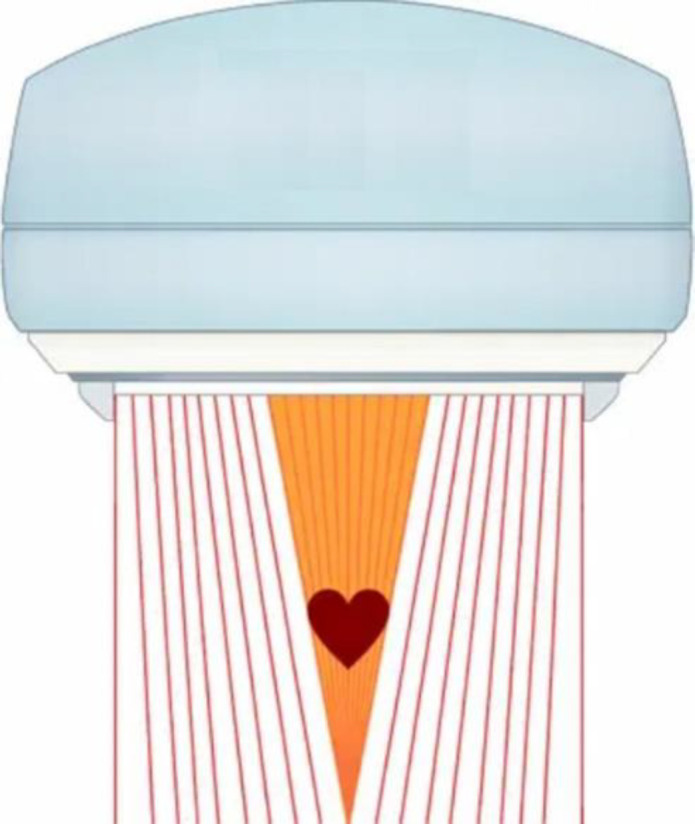
SMARTZOOM(TM) collimator – cardio-centric orbit Configuration of SMARTZOOM(TM) collimator with converging collimator at the centre and parallel whole collimator at the periphery

 Cardiac-specific collimators have been introduced, which have improved the acquisition as well as the image quality in MPI. IQ-SPECT technology (Siemens Medical Solutions USA, Inc., Hoffman Estates, IL, USA) uses cardiac specific SMARTZOOM(TM) collimator, which has a combination of a converging collimator at the center and a parallel-hole collimator at the periphery (Figure 1). This technology possesses three critical qualities, which include smart zoom collimation, cardio-centric orbit, and special iterative reconstruction methods. The addition of an SMARTZOOM(TM) collimator has shortened the acquisition time, producing an image of higher quality and resolution than the routinely used LEHR collimators (3).

 The standard acquisition time of MPI with the LEHR collimator is approximately 16 minutes (32 views, with 30 seconds per view). In contrast, acquisition with the SMARTZOOM (TM) collimator is shorter, with a duration of 4 minutes (17 stops per detector at 14 seconds per stop). The image quality is comparable, and the SMARTZOOM(TM) collimator exhibits the added advantages of faster acquisition and higher sensitivity (4). However, even a 4- minute duration of imaging might be uncomfortable for certain patients because of the prolonged positioning of hands above the head, especially for those with claustrophobia and musculoskeletal disorders. Thus, this study aimed to shorten the scan acquisition time further using the SMARTZOOM(TM) collimator for MPI without compromising the image quality and attempted to analyze the effects of shortening the time per stop to 7 seconds. 

## Methods

 A prospective observational study was conducted in the Department of Nuclear Medicine, Apollo Hospitals, Chennai, over a period of 2 years from 2019 to 2022. This study involved 50 participants (39 men and 11 women) in the age group of 39–81 years (mean age 56.32 years). Patients referred to MPI for the evaluation of CAD were included in the study. Patients who were reluctant to lie down for a long duration (for two scans) were excluded. Patients with bariatric issues who had a body mass index of ≥30 were not included in the study as the standard dose and a scan acquisition time of 8 minutes are recommended by the manufacturer (SIEMENS) for this patient subgroup. This study was approved by the institutional review board, and written informed consent for participation in the study was obtained from all patients. The patients were instructed to observe 2 hours of fasting before the procedure.

 Rest and stress MPI were performed under a 22-day protocol. The mode of stress (physical or pharmacological) was decided by the nuclear medicine physicians as indicated. A single injection of 10–12 mCi (370–444 MBq) Tc-99m MIBI was administered intravenously at rest and peak stress (5). Milk-based fat-rich blend, which was prepared in-house, was given to the patients post-injection to enhance the hepatic clearance of the tracer. MPI was performed at 60–90 minutes post-injection in a dual-headed gamma camera with cardiac-specific SMART ZOOM(TM) collimators (IQ-SPECT, Symbia T6, SIEMENS healthcare). The manufacturer’s recommendation for MPI acquisition using the SMARTZOOM(TM) collimator in a dual-headed gamma camera is 17 stops per detector at 14 seconds per stop, thus resulting in a total of 34 projections acquired in 4 minutes.

 The participants underwent rest and stress MPI scan acquisitions in a dual-headed gamma camera with the SMARTZOOM(TM) collimator-each with half the recommended acquisition time (7 seconds per stop) as well as the standard acquisition time (14 seconds per stop). Thus, two rest and two stress MPI studies were acquired for each patient (6-8). The image acquisition parameters are listed in Table 1. A low-dose CT acquisition for attenuation correction followed the SPECT acquisition.

**Table 1:** Acquisition parameters for MPI

Position of the detectors	76⁰
Collimator type	SMARTZOOM(TM) collimator
Angle of rotation	104⁰
Stops/Views	17 per detector
Duration per stop	7 seconds and 14 seconds
Matrix size	64×64
Reconstruction algorithm	Iterative Flash 3D
Position of the patient	Supine
ECG gating	Gate bin 8
Number of frames	8

 The MPI raw data were processed using the Cedar Sinai software. After reconstruction, the images were reproduced in three axes—short axis, vertical long axis, and horizontal long axis—and analyzed both qualitatively (visual inspection based on the color scale) and quantitatively using automatic quantification, i.e., quantitative-gated and perfusion SPECT (QGS/QPS). Polar maps with 17 segments were generated for the perfusion analysis. Gated images were used to analyze the left ventricular wall motion and thickening for all scans.

 The rest and stress image datasets of the study population were categorized into two groups, Group A (7 seconds) and Group B (14 seconds), and were scrutinized with respect to image quality, relative uptake score, summed scores, and total perfusion deficit (TPD). Furthermore, the left ventricular ejection fraction (LVEF) was compared between the two groups for the quantitative assessment (9, 10).

***Image quality scoring***

 Two experienced nuclear medicine physicians assessed the image quality using the 17-segment cardiac model of the American Society of Nuclear Cardiology (ASNC). For subjective evaluation of the image appearance (Table 2) between the two groups, an ordinal score of 1–5 was assigned for each entire image set of the left ventricular myocardium. 

**Table 2:** Image quality score

**Image quality score**	
1	Very poor
2	Poor
3	Adequate
4	Good
5	Very good

***Relative uptake scoring***

 The scores given by the software for the relative activity in each segment of the myocardium compared with the images of the normal database (11) are presented in Table 3.

**Table 3:** Relative uptake score

**Relative uptake score**	
0	Normal perfusion
1	Mild reduction in counts but definitely not abnormal
2	Moderate reduction in counts and definitely abnormal
3	Severe reduction in counts
4	Absent tracer uptake

 Statistical analysis was performed using SPSS (Statistical Package for the Social Sciences) version 18.0.

### Camera parameter settings for IQ-SPECT with SMARTZOOM(TM) collimator

 Zoom: 1.0; Matrix 64×64; 17 views per detector obtained at 7 seconds and 14 seconds per view. A scanning arc of 104⁰ over angular steps of 6⁰ was used for each of the camera heads.

 The rotation was restricted to 104⁰ to keep the heart 28 cm from the camera surface throughout the acquisition to maximize the sensitivity. The ECG gating threshold was 20%, and the gate bin used was 8. Gated at 8 frames per cardiac cycle, neither attenuation nor scatter correction was applied.

 Cedars Sinai QGS and QPS analysis: The reconstruction was performed using the Flash 3-dimensional ordered subset conjugate gradient minimize (3D OSCGM) algorithm, 30 iterations with 1 subset, and a Gaussian filter with a full-width half maximum (FWHM) of 14 mm.

 Both attenuation and scatter correction were applied for Tc-99m. Both acquisitions were obtained for all participants (7 seconds/14 seconds).

## Results

Group A and Group B images were compared in terms of LVEF, summed scores, TPD, relative uptake score, and image quality. Pearson values were analyzed for quantitative and qualitative assessment in both groups. A p-value of <0.05 was considered statistically significant.

### LVEF

 The comparison of LVEF between Groups A (7-second images) and B (14-second images) did not show any significant difference between the two datasets. The mean LVEF values were 62.16 and 64.34 in Group A (7-second images) and Group B (14-second images), respectively (p-value 0.326). A strong positive correlation was observed between the two datasets (Pearson’s r = 0.59).

### Summed scores

 The total summed score was calculated for the left ventricular myocardium at rest and stress for each group. Significant differences were not noted in the summed scores between the two groups. The mean summed score for attenuation-corrected (AC) images was 7.5 in Group A and Group B (p-value 0.49), and those for non-attenuation-corrected (NAC) images were 7.68 in Group A and 7.46 in Group B (p-value 0.46).

### TPD

 The TPDs for both groups were calculated according to the 17-segment cardiac model of the ASNC guidelines in both AC and NAC studies. Significant differences were not perceived between the two groups in both AC and NAC images. The mean TPDs were 11% and 16% in Groups A and B, respectively, for AC images (p-value 0.143) and 11.2% and 16.5% in Groups A and B, respectively, for NAC images (p-value 0.135).

 A strong correlation was observed between Groups A and B in the calculation of TPD using the AC and NAC images (AC Pearson r=1; NAC Pearson’s r=1).

### Relative uptake score

 The calculation of the relative uptake score in segments 1–17 for AC and NAC images in Groups A and B did not show significant differences. 

### Image quality

 The counts per frame were in the range of 24000–27000 for Group A (7 seconds) and 48000–52000 for Group B (14 seconds). Both Group A (7 seconds) and Group B (14 seconds) images were of comparable quality in the AC studies, whereas Group B was superior to Group A in the NAC images. However, all images had an image quality score of ≥3, which suggested adequate overall image quality.

 Thus, the use of the IQ-SPECT technology with SMARTZOOM(TM) collimator, cardio-centric orbit, iterative reconstruction method, and CT-based attenuation correction could reduce the acquisition time while maintaining comparable image quality.

## Discussion

IQ-SPECT technology has a high-efficiency multi-cone focal SMARTZOOM(TM) collimator, cardio-centric orbit, iterative reconstruction, and scatter correction. These notable features aid in achieving better scan quality with shorter acquisition time in MPI when compared with the LEHR collimator (12). Hence, further reduction of acquisition time from 14 seconds per view to 7 seconds per view is feasible with IQ-SPECT technology.

 Leva L et al. conducted a comparative study in patients who had undergone MPI using both LEHR and SMARTZOOM(TM) collimators regarding the tolerance and comfort levels. The findings indicated that patients were more comfortable with studies done in SMARTZOOM(TM) collimators than in LEHR collimators as the acquisition time was reduced by half (13). The acquisition times per angular step of the detector using SMARTZOOM(TM) collimators were 14 and 28 seconds/view in their study. In this study, the feasibility of further reducing the acquisition time to half using SMARTZOOM(TM) collimators (i.e., 7

 seconds/view) without compromising the image quality to improve patient comfort was analyzed.

 Parameters such as image quality, LVEF, summed score, TPD, and relative uptake score were used as standards to compare the images acquired in 7 seconds per view (Group A) and 14 seconds per view (Group B) using the SMARTZOOM(TM) collimator.

 Caobelli et al. compared myocardial perfusion studies performed using IQ-SPECT technology in 6 seconds per view and 12 seconds per view and concluded that the image quality was reduced in case of shorter acquisition time (7). 

 However, our findings indicated comparable image quality between studies done in 7 seconds per view and 14 seconds per view in AC. All images exhibited quality scores of ≥3, which suggests adequate overall image quality.

**Figure 2:**
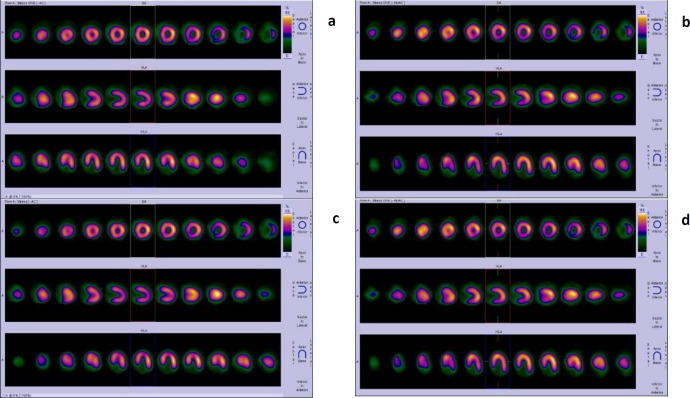
52-year-old male with diabetes, recently detected coronary artery disease, 70% stenosis in left anteriordescending artery (LAD), was referred for myocardial perfusion imaging to assess the provocable ischemia in LAD territory. Both rest and physical stress myocardial perfusion studies were performed. Attenuation-corrected (AC) and non-attenuation-corrected (NAC) stress images were reconstructed in both 7 seconds and 14 seconds per view (Figures **a**, **b**, **c** and **d**, respectively) (**a**) 7 seconds AC image, (**b**) 7 seconds NAC image, (**c**) 14 seconds AC image, (**d**) 14 seconds NAC images

**Figure 3:**
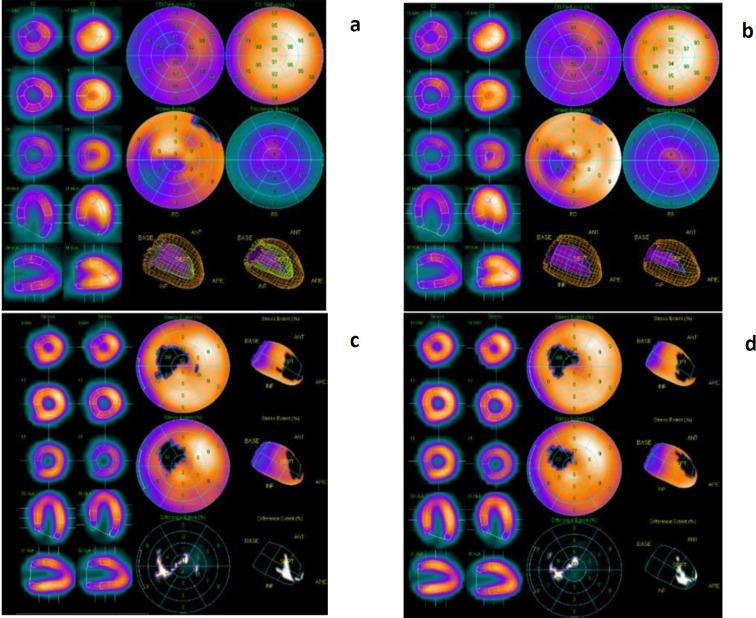
A 48-year-old male with a history of acute myocardial infarction, Triple vessel disease, post PTCA to left anterior descending artery (LAD) was referred for myocardial perfusion imaging 3 months later to assess provocable ischemia in left circumflex (LCx) and right coronary artery (RCA) territories. Both rest and physical stress myocardial perfusion studies were performed. Stress study was reconstructed in both 7 seconds and 14 seconds per view using the automatic quantification QGS and QPS (Figures **a**, **b**, **c** and **d**, respectively). Polar maps with 17 segments were generated for the perfusion analysis. (**a**) QGS 7 seconds/ view,(**b**) QGS 14 seconds/ view, (**c**) QPS 7 seconds/ view, (**d**) QPS 14 seconds/ view

 In this study, no significant difference was observed in the summed score results, TPD, or LV volume between the studies done in 7 seconds per view and 14 seconds per view. Moreover, a strong positive correlation was observed in the LVEF between the studies done in 7 seconds per view and those done in 14 seconds per view (representative images: Figures 2 and 3).

**Table:** All the images (Figures 2a, 2b, 2c and 2d) were of comparable quality. The qualitative and quantitative comparison parameters in both groups A and B were as follows:

	**Group A**		**Group B**	
	**AC images**	**NAC images**	**AC images**	**NAC images**
Image quality score	5	5	5	5
Relative uptake score	0	0	0	0
Summed stress score	0	0	0	0
TPD	0	0	0	0
LVEF	66		67	

**Table:** All the images (Figures 3a, 3b, 3c and 3d) were of comparable quality. The qualitative and quantitative comparison parameters in both group A and B were as follow:

	**QGS**		**QPS**	
	**Group A**	**Group B**	**Group A**	**Group B**
Image quality score	5	5	5	5
Summed stress score			11	11
TPD			10	8
LVEF	69	70		

Our study, therefore, demonstrated a strong correlation in parameters such as image quality, TPD, summed score, and LVEF using SMARTZOOM(TM) collimator with a standard dose and shortened acquisition time. This could be attributed to the collimator’s indigenous ability to magnify the center of the field of view (i.e., the heart in the myocardial perfusion study) while avoiding truncation from the periphery.

**1. SMARTZOOM(TM) collimator:** The focus of the collimator varies continuously from cone-like geometry in the center to parallel geometry toward the periphery, thus providing magnification of the cardiac region while avoiding the truncation of the surrounding tissue. Unlike conventional large-bore, parallel-hole collimators, the SMARTZOOM(TM) collimator achieves a gain in counts without compromising the image resolution, reducing the imaging time considerably. 

**2. Cardio-centric acquisition:** IQ-SPECT technology is superior to conventional scan acquisition owing to its software-assisted flexible gantry motion feature. The cardio-centric orbit allows the detectors to rotate around a virtual center of rotation positioned over the heart. The characteristic features of this centric acquisition are the relative detector position, the radius of rotation, and the arc used to acquire images of the heart from all view angles. These characteristics aid in increasing the sensitivity and resolution.

**3. Reconstruction:** IQ-SPECT reconstruction includes the geometry of SMARTZOOM(TM) collimators, cardio-centric orbit of the detectors, and ordered subset expectation maximization reconstruction. This reconstruction technique also comprises state-of-the-art distance-dependent isotropic 3D resolution recovery and ordered subset conjugate gradient minimizer (14).

## Conclusion

 A 7-second per view acquisition is possible in MPI and has image quality similar to a 14-second per view acquisition using the SMARTZOOM (TM) collimator in IQ-SPECT technology. Thus, IQ-SPECT technology enables the acquisition of comparable-quality scans in a shorter time with a standard dose to assess TPD, summed score, and LVEF without the loss of any diagnostic information. Faster scan acquisition is possible, thus reducing the waiting time of patients, increasing the department’s efficiency, and streamlining the workload. In addition, scan acquisition can be performed with ease in patients having claustrophobia and debilitating conditions without compromising their comfort.
